# Customizable automated cleaning of multichannel sleep EEG in SleepTrip

**DOI:** 10.3389/fninf.2024.1415512

**Published:** 2024-08-09

**Authors:** Roy Cox, Frederik D. Weber, Eus J. W. Van Someren

**Affiliations:** ^1^Department of Sleep and Cognition, Netherlands Institute for Neuroscience, Amsterdam, Netherlands; ^2^Donders Institute for Brain, Cognition and Behavior, Nijmegen, Netherlands; ^3^Departments of Integrative Neurophysiology and Psychiatry, Center for Neurogenomics and Cognitive Research, Amsterdam University Medical Center, Amsterdam Neuroscience, VU University, Amsterdam, Netherlands

**Keywords:** EEG, sleep, polysomnography, artifacts, software

## Abstract

While standard polysomnography has revealed the importance of the sleeping brain in health and disease, more specific insight into the relevant brain circuits requires high-density electroencephalography (EEG). However, identifying and handling sleep EEG artifacts becomes increasingly challenging with higher channel counts and/or volume of recordings. Whereas manual cleaning is time-consuming, subjective, and often yields data loss (e.g., complete removal of channels or epochs), automated approaches suitable and practical for overnight sleep EEG remain limited, especially when control over detection and repair behavior is desired. Here, we introduce a flexible approach for automated cleaning of multichannel sleep recordings, as part of the free Matlab-based toolbox SleepTrip. Key functionality includes 1) channel-wise detection of various artifact types encountered in sleep EEG, 2) channel- and time-resolved marking of data segments for repair through interpolation, and 3) visualization options to review and monitor performance. Functionality for Independent Component Analysis is also included. Extensive customization options allow tailoring cleaning behavior to data properties and analysis goals. By enabling computationally efficient and flexible automated data cleaning, this tool helps to facilitate fundamental and clinical sleep EEG research.

## 1 Introduction

Sleep electroencephalography (EEG) is the primary method for investigating the human sleeping brain in health and disease. EEG signals are the main component of polysomnography (PSG), the gold standard for defining sleep and wake states (Rechtschaffen and Kales, 1968; Berry et al., 2018). Beyond its fundamental role in sleep staging, sleep EEG may undergo numerous signal processing and analysis steps to reveal the complex dynamics and functions of sleep (Cox and Fell, 2020; Hermans et al., 2022). Both lab- and home-based EEG systems with few channels (e.g., 2–10) are increasingly being leveraged for large-scale examinations across hundreds or thousands of recordings (Purcell et al., 2017; Kozhemiako et al., 2022). At the same time, many research and clinical labs routinely acquire high-density EEG recordings (e.g., 32–256 channels) to reveal important topographical aspects of sleep dynamics, clinical disorders, and sleep functions (Pisarenco et al., 2014). However, as sleep EEG studies increase in scale, monitoring and safeguarding data quality become more challenging.

Obtaining valid insights from sleep EEG requires analyses be performed on signal portions minimally affected by non-neural activity, as such artifacts can heavily influence results. Sleep EEG artifacts can be physiological or technical in nature, and include eye movements and blinks, sweat artifacts, muscle twitches, large body movements, arousals, electrolyte evaporation and bridging, signal discontinuities, amplifier saturation or disconnection, cardiac and pulse activity, respiratory activity, swallow artifacts, and likely others (Anderer et al., 1999; Attarian and Undevia, 2012). Note, however, that some of these event types could themselves be targets of inquiry (e.g., rapid eye movements, arousals). Although the importance of data cleaning is generally acknowledged, identifying and handling artifacts in sleep EEG is particularly challenging due to data length and the many possible artifact types that may have distinct or varying patterns of topographical expression across the night.

To date, most cleaning approaches include a large manual component, involving visually marking segments of fixed length (typically 30-s epochs) for rejection, and/or marking entire channels for rejection or repair. Besides being time-consuming, subjective, and poorly reproducible, complete removal of channels and epochs is wasteful given that artifacts are often expressed in only a subset of channels or for a limited time. While artifacts could conceivably be manually marked on a per-channel basis, this is seldom done in practice even with low channel or recording numbers, let alone for a large volume of high-density EEG recordings.

Despite the drawbacks of manual cleaning, automated approaches for handling sleep EEG artifacts are neither commonplace nor consistent in sleep research. Although a wide variety of signal processing algorithms exist for detecting EEG artifacts during wakefulness (Jiang et al., 2019), many of these detect artifacts globally (i.e., not channel-resolved) and address only one or a few artifact type(s). As such, these methods are generally not suitable for sleep EEG with its unique physiological substates and numerous, spatially evolving, artifact types. Moreover, many algorithms developed in the context of wake EEG are computationally intensive, making unreasonable memory demands or having impractically long runtimes for an 8-h overnight high-density recording. Perhaps most importantly, low-level stand-alone detection algorithms are of little practical use to most users in the absence of additional functionality for data handling, repair, and visualization. On the other hand, various high-level toolboxes suitable for EEG, such as FieldTrip (Oostenveld et al., 2011), EEGLAB (Delorme and Makeig, 2004), Brainstorm (Tadel et al., 2011), and MNE-Python (Gramfort, 2013), provide powerful data processing and analysis utilities, including various artifact detection and repair options. However, these platforms are also geared heavily towards wake EEG, which is typically much shorter and homogeneous than sleep EEG, and these tools contain very limited built-in solutions suitable for sleep.

A relatively small number of dedicated sleep EEG cleaning approaches have been reported (although it is likely that various “in-house” pipelines have gone unpublished). Two of these approaches are limited to the detection of pre-specified global artifact types and lack the functionality for further handling these events [FASST (’t Wallant et al., 2016); Riemannian Potato (Saifutdinova et al., 2019)]. In contrast, High-Density-SleepCleaner offers channel- and epoch-resolved artifact detection, including important visualization and epoch-wise interpolation options (Leach et al., 2023). A drawback of this approach is that it relies on experienced users to visually screen each recording multiple times. The Luna toolbox (Kozhemiako et al., 2022) includes fully automated epoch- and channel-resolved flagging of outliers and several artifact detection and repair options, including epoch-wise interpolation. Finally, SleepEEGpy (Belonosov et al., 2023) enables visual annotation of bad channels and temporal intervals, but specific artifact detection routines are not included. Table 1 compares various features for the aforementioned toolboxes.

**TABLE 1:** Feature comparison of various toolboxes and algorithms relevant for sleep EEG artifact processing.

Toolbox/ algorithm	Language (dependency)	Suitable for high-density sleep EEG	Artifact type(s)	Artifact detection	Artifact visualization	Interpolation functionality
**high-level toolboxes**						
EEGLAB	Matlab	Yes	Many, customizable	Global	Global	Recording-wise
FieldTrip	Matlab	Yes	Many, customizable	Global	Global	Recording-wise
Brainstorm	Matlab (standalone)	Unknown	Limited, customizable	Global	Global	Recording-wise
MNE-Python	Python	Unknown	Limited, customizable	Global	Unknown	Recording-wise, epoch-wise
**Low-level toolboxes**						
Luna	C/C++/R	Likely	Many, customizable	Channel-wise	Unknown	Epoch-wise
FASST	Matlab	Possibly	Fixed	Global	No	No
Riemannian potato	Python	Possibly	Fixed	Global	No	No
High-Density-SleepCleaner	Matlab	Yes	Fixed	Channel-wise	Channel-wise	Epoch-wise
SleepEEGpy	Python (MNE-Python)	Yes	Unknown	Unknown	Unknown	Recording-wise, epoch-wise
current	Matlab (SleepTrip)	Yes	Many, customizable	Channel-wise	Channel-wise	Epoch-wise

Information compiled from publications and other available documentation, and may not reflect current functionality.

Blind source separation methods, such as independent component analysis (ICA), represent another approach to removing artifacts from sleep data (Romero et al., 2003; Crespo-Garcia et al., 2008; Demanuele et al., 2017). Rather than detecting individual events with specific start and end times, these approaches decompose multichannel EEG time courses into component time courses, some of which usually correspond strongly to observable artifacts (e.g., ocular, cardiac) and may be mathematically subtracted from the original signal. However, manual component selection requires additional human expertise and is difficult to replicate, while the resulting data invariably contain residual artifacts that require handling. Hence, while ICA may be considered a useful preliminary cleaning step, it rarely yields analysis-ready data.

In sum, limited practical solutions exist for users wishing to set up automated artifact handling for sleep EEG, particularly when cleaning is to be tailored to specific data properties or analysis goals. Drawing inspiration from existing tools and algorithms, we here introduce a flexible approach to automated cleaning of overnight sleep recordings as part of SleepTrip (Weber et al., 2021), a toolbox suitable for large-scale sleep EEG analyses. Functionality includes channel-resolved detection of various artifact types encountered in sleep EEG, followed by both the marking of data segments for complete rejection (i.e., ignore or remove), and temporally resolved repair of artifactual channels via interpolation. In addition, pre-cleaning ICA may further improve data quality. The approach is computationally efficient, suitable for all channel counts, and offers extensive customizability to align cleaning behavior with analysis goals. Finally, this tool may serve as a basis for creating standardized cleaning pipelines, thereby improving data analyses and interpretation.

## Methods and results

### Overview

The present cleaning functionality is part of the free Matlab-based toolbox SleepTrip,^1^ allowing artifact handling to be integrated into a large suite of functions for data handling and process pipelining, preprocessing, and analysis (e.g., sleep architecture, spectral power, sleep spindle and slow wave detection). As SleepTrip is a branch of FieldTrip (Oostenveld et al., 2011), the present approach relies on and repurposes various FieldTrip functions, as well as some EEGLAB functionality (Delorme and Makeig, 2004) including the IClabel plugin (Pion-Tonachini et al., 2019). Tutorial scripts to get started with artifact detection (*tutorial_cleaning.m*) and ICA (*tutorial_ica.m*) are included within SleepTrip. Three accompanying 64-channel recordings can be downloaded from within SleepTrip (*download_tutorial_data.m*) or accessed directly from Zenodo.^2^

### Requirements

SleepTrip runs under Matlab (development versions: R2022a and R2022b for Windows) with access to the Signal Processing Toolbox. Hardware requirements depend on recording characteristics: a 8-h 251-channel recording sampled at 250 Hz has a peak memory usage of ∼128 GB RAM and a runtime of ∼45 min on a 3.60 GHz 4 core machine, with these values scaling roughly proportionally with channel count, recording length, and sample rate. Approximate runtimes using our setup for individual cleaning components and ICA are reported in the respective sections.

Data cleaning requires three primary variables as input:

1) *data*: a FieldTrip structure containing sleep EEG data, with underlying data requiring 64-bit precision (Matlab’s *double* type) for signal processing. *data* should contain only continuous signal (no trials/epochs) provided in the shape desired for further processing (e.g., desired channel and time range, reference, filter). Unless otherwise required, sample rates above 250/256 Hz are discouraged to ensure acceptable runtimes.
2) *elec*: a structure containing electrode/channel coordinates, in FieldTrip format. *elec* should match the channels contained within *data*. *elec* is used for determining neighboring channel pairs, as used by several artifact detectors, and for making topographical plots.
3) *scoring*: a structure containing sleep stage information, in SleepTrip format.

Various SleepTrip and FieldTrip resources and functions assist in preparing these variables as required.

### Dataset

The cleaning tool was developed in conjunction with an existing dataset consisting of 560 256-channel overnight recordings from 371 individuals (47.4 ± 13.7 y, 261 female), with each individual contributing between 1 and 5 recordings. Data stems from different study protocols, with individual study protocols approved by the ethics committees of either VU Medical Center or University of Amsterdam. Participants gave written informed consent in accordance with the Declaration of Helsinki, and were paid for participation. Participants were patients diagnosed with insomnia disorder (N = 245), healthy sleepers (N = 120), unclassified pilot participants (N = 5), and a patient diagnosed with advanced sleep phase disorder (N = 1). Based on previous data examination, this sizable and heterogeneous sample was known to harbor large variability regarding artifact type, severity, and spatiotemporal expression. EEG was sampled at 1,000 Hz with an online Cz reference, along with several physiological bipolar signals (Electrical Geodesic Inc., Eugene, OR, USA). Offline, EEG was downsampled to 250 Hz, rereferenced to digitally linked mastoids, and filtered (high-pass at 0.5 Hz, notch at 50 Hz). Final channel count was 251, but recording variants with fewer channels were also examined. Separately, recordings were converted to a PSG montage of standard EEG, electrooculography (EOG) and electromyography (EMG) for manual scoring of sleep stages and arousals (Berry et al., 2018).

### Artifact detection and repair

**FIGURE 1:**
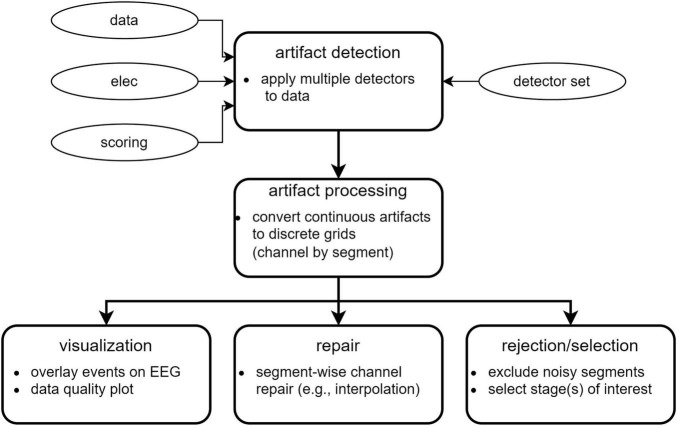
Overview of data cleaning steps.

Figure 1 illustrates the conceptual workflow of the main artifact detection and repair functionality. Optional ICA-based pre-cleaning is described later.

#### Artifact detection

To enable artifact detection of various types, the user supplies a so-called *detector set* comprised of individual detectors. A default detector set, presently containing nine individual detectors, may be used as a starting point (Table 2). Default detectors’ settings may be altered or new detectors may be constructed to build a custom detector set optimized for the data at hand. Each individual detector contains low-level instructions regarding data to consider (sleep stages and channels), how data should be processed (e.g., filtering, Hilbert transform, smoothing, z-scoring), and detection criteria (e.g., amplitude and duration thresholds, merging rules). When running a detector set, each individual detector is applied either separately to each channel, or to designated channel pairs, depending on detector type. Specifically, channel-wise detectors identify signal portions meeting the specified requirements separately on each channel (e.g., the default *highamp* detector). In contrast, pairwise detectors operate on fixed-length windows (typically 30 s) and calculate a pairwise metric (e.g., correlation) for all channel pairs involving a particular target channel. The target channel is then labeled artifactual if a sufficient proportion of values meets criteria (e.g. for the default *deviant* detector: unrealistically low correlation with the majority of local neighbors). Channel pairs to consider are specified by a customizable neighborhood structure (e.g., local neighbors or all-to-all).

**TABLE 2:** Overview of individual artifact detectors contained within the default detector set.

Detector	Artifact type(s)	Entity	Method	Minimum duration (s)	Padding (s)	Merge interval (s)	Runtime 6/64/250 (min)	Notes
Highamp	Excessive positive/negative amplitude	Channel-wise, continuous	Absolute amplitude > 300 μv	0	0.1	1	<1 <1 ∼2	
Lowamp	Implausibly low amplitude	Channel-wise, continuous	Absolute amplitude < 5 μv	30	0.1	1	< 1 <1 ∼2	
Lowfreq	Excessive low-frequency activity (e.g., ocular, sweat)	Channel-wise, continuous	0.3–15 Hz signal envelope; z > 8	0	3	1	<1 ∼2 ∼8	
Highfreq	Excessive high-frequency activity (e.g., muscle)	Channel-wise, continuous	60–120* Hz signal envelope; z > 3	0	0.1	1	<1 ∼2 ∼8	*:lowered to nyquist if sample rate < 240 Hz; dropped at 80 Hz
Jump	Signal jumps (e.g., channel pops)	Channel-wise, continuous	Median filter (order 9); absolute gradient; Z > 25	0	0.1	1	<1 <1 ∼2	Filter order for 250 Hz
Flatline	Unchanging signal (e.g., no connection, saturation)	Channel-wise, continuous	Absolute gradient < 1 μv	1	0.1	1	<1 <1 ∼2	Threshold adjusted based on sample rate (e.g, 2 μv for 125 Hz)
Deviant	Channel deviates strongly from local neighbors (e.g., poor/no connection)	Pairwise (local), window (30 s)	R with neighbor < 0.3 for > 0.5 of neighbors	[window length]	0	60	<1 <1 <1	Only if > 2 channels
Similar	Channel highly similar to local neighbors (e.g., gel bridging)	Pairwise (local), window (30 s)	Maximum absolute difference between neighbors < 0.5 μv	[window length]	0	60	<1 <1 ∼15	Only if > 2 channels
Similar2	Channel highly similar to majority of channels (e.g., poor reference)	Pairwise (global), window (5 s)	High-pass 2 Hz, R of chan pair > 0.9 for > 0.5 of neighbors	[window length]	0	5	<1 <1 ∼2	

Default detectors and their algorithmic details may be subject to change.

All detectors return event tables with event start and end times by channel, to be used both for downstream processing (see *Artifact processing*) and event visualization (see *Artifact visualization*). Total runtime using the default detector set is approximately 1, 10, and 45 min for channel counts of 6, 64, and 250, respectively.

#### Artifact processing

For each detector, its event table is converted into a binary channel-by-segment artifact grid (default segment length: 5 s). Here, a grid element is labeled artifactual if the corresponding continuous data segment is artifactual for more than a certain proportion of time (default: 0). The discrete grid format simplifies further processing, while the short segment length results in limited loss of granularity.

Following the conversion to a grid-based representation, additional processing proceeds in five steps. First, individual detector grids are pooled (default: logical *OR* across all detector grids) into a *basic artifact grid*, indicating whether channel-segment elements contain artifact of any kind. Grid merging may also be performed on a subset of available detector grids. Second, a *spatial expansion grid* is created: at each segment, channels not containing basic artifact are set to artifactual if a sufficient proportion of neighboring channels is artifactual. This step is particularly suitable for high-density recordings, marking channels surrounded by artifactual channels but failing to reach detection thresholds themselves. By default, no spatial expansion is performed. Third, a *rejection grid* is created: segments where the proportion of artifactual channels is deemed too high are marked for rejection (i.e., downstream removal). By default, no segments are rejected. Fourth, a *temporal expansion grid* is created by identifying channels where the proportion of artifactual segments is deemed too high, and extending artifacts to all segments of the affected channels. Hence, this step essentially functions as a bad channel detector. Note that this step occurs after the *rejection grid* has been created, such that bad channel detection is not driven by segments already marked for rejection. By default, no temporal expansion is performed. Finally, and fifth, the *repair grid* marks channel-segment elements for downstream repair. It is created by combining all grids except the *rejection grid* (i.e., *basic artifact grid*, *spatial expansion grid*, *temporal expansion grid*). The *rejection grid* is then subtracted from the *repair grid*, as segments marked for rejection typically contain too many noisy channels to allow meaningful repair.

Next, various summary statistics are calculated from the grids, including percentage of data marked artifactual for individual artifact grids, and overall percentages marked for repair and rejection. Statistics are also provided by sleep stage. This information can be used to identify recordings requiring additional review or to discard entirely. Grids are also converted back to event tables for viewing them in relation to EEG traces (see *Artifact visualization*). Artifact processing runtime is typically < 1 min.

#### Artifact visualization

**FIGURE 2:**
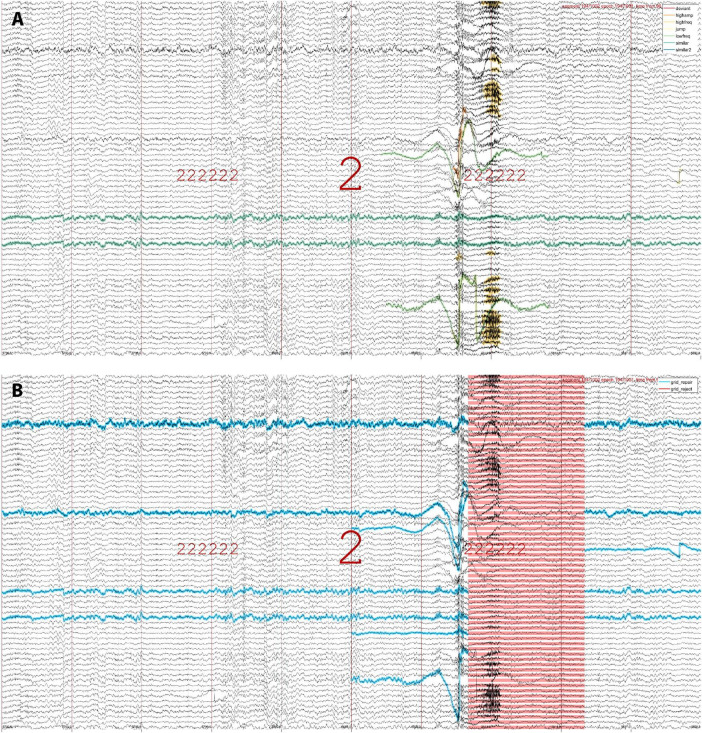
Event visualization. **(A)** An epoch of N2 (indicated by large 2) both preceded and followed by six N2 epochs (sequence of small 2 s). Raw artifacts of various types are highlighted in different colors (legend in top-right), including instances of high-amplitude, low-frequency, high-frequency, jump, deviant-channel, and similar-channel artifacts. Note that different artifact types may overlap, thereby building in some redundancy. Also note the two channels marked as similar (green-blue): these channels were numerically identical across the entire recording but would likely not be flagged manually. **(B)** The same epoch as in **(A)**, now showing channel-segment elements marked for repair (blue) and segments marked for rejection (red). Note that the top channel labeled for repair does not contain artifacts within this epoch, but it is marked due to its poor quality throughout the rest of the recording. Artifact detection run on 251 channels but only 70 shown for visualization purposes.

To examine whether detected events reflect visually apparent artifacts, SleepTrip offers low-level event visualization functionality. Specifically, event tables generated by both raw artifact detection and subsequent grid-based artifact processing may be used to overlay events on top of EEG traces and scroll through the recording for visual review. As an example, Figure 2A highlights raw artifacts of various types for an epoch of N2. Figure 2B shows the same data while highlighting the grid elements marked for repair (blue) and rejection (red). Event tables produced by different detectors may be combined or shown in isolation, results from detectors with different settings may be compared, and events may be overlaid on pre- or post-cleaning data. Event tables are not strictly limited to artifacts and could also contain other events (e.g., spindles, slow waves, eye movements). Color and marking style for each event type are customizable.

**FIGURE 3:**
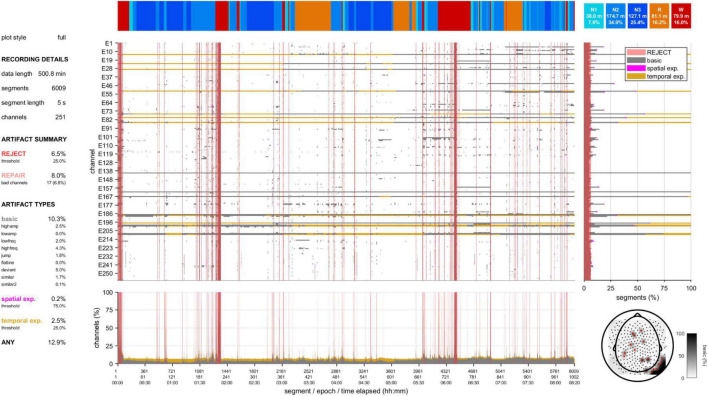
Data quality plot for the recording containing the epoch shown in Figure 2. The data quality plot shows descriptive information on rejection, repair, and artifact percentages (left), a hypnogram (top), a central grid indicating channel-segment elements marked as artifact (gray: basic artifact, magenta: spatial expansion, gold: temporal expansion) and segments marked for rejection (red), marginal summaries of the central grid across channels (bottom) and across segments (right), and a topography (bottom-right) of basic artifact percentages (white-black gradient) and channels marked for complete interpolation (red).

A second visualization tool provides a high-level data quality plot containing the most important aspects of artifact detection, including many of the aforementioned grid-based summary statistics, various overlaid grids, a topography, and a hypnogram (Figure 3 and Supplementary Figures 1–6). This information can be helpful to provide an immediate impression of data integrity without scrolling through an entire recording. At the same time, it can signify parts of the data requiring more detailed review.

#### Data repair

**FIGURE 4:**
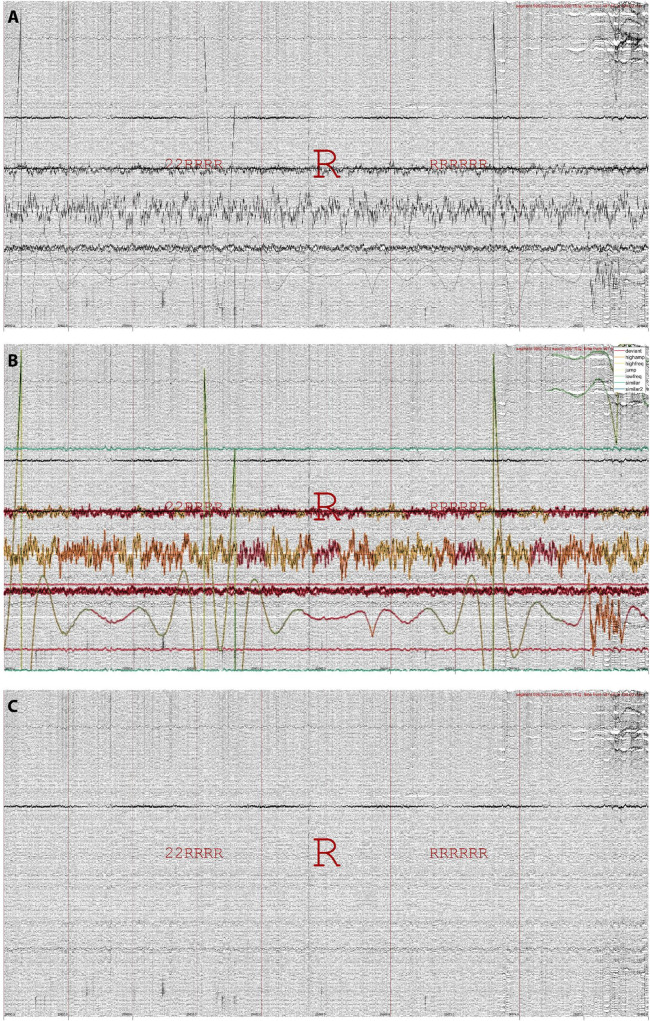
Data repair using segment-wise channel interpolation in 251-channel data. **(A)** An epoch of REM sleep (R, both preceded and followed by mostly REM) in the last quarter of the night, when various channels have become noisy or disconnected entirely. **(B)** The same epoch with detected artifacts highlighted. **(C)** The same epoch after segment-wise channel interpolation. Note the overall improvement in signal appearance, although some data disturbances remain.

Data may be repaired in several ways, typically by making use of the *repair grid*. The primary repair method is by interpolating artifactual channels using the weighted spherical spline approach (Freeden, 1984; Oostenveld et al., 2011). Importantly, data repair proceeds segment-wise, such that at each segment only those channels labeled artifactual are repaired, using exclusively information from non-artifactual channels. Discontinuities arising from this approach are minimized by smoothing affected segment boundaries. Figure 4 illustrates data repair for a 251-channel epoch of REM sleep. Alternatively, the repair grid can be used to replace affected channel-segment elements with a fixed value (zero or not-a-number). It is also possible to base data repair on any of the other built-in grids, or on a custom grid (e.g., calculated from existing grids and/or including manual edits). The data repair routine typically takes < 1 min.

#### Data rejection and selection

**FIGURE 5:**
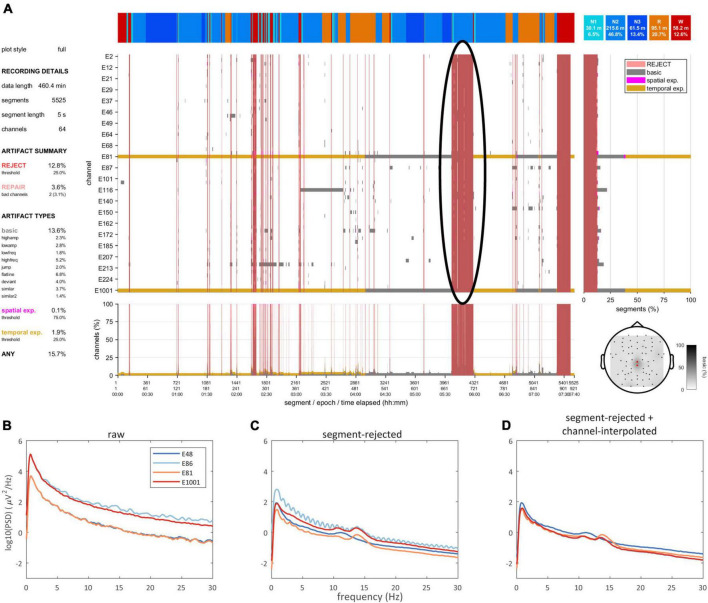
Data quality and spectra. **(A)** Data quality plot showing substantial artifact during late N2, which is marked for rejection (black oval). Power spectra of selected channels during N2 for raw **(B)**, segment-rejected **(C)**, and segment-rejected plus channel-interpolated data **(D)**.

A data selection routine allows excluding data marked for rejection. Data may be returned in trial format (i.e., separating each uninterrupted data bout) or pseudocontinuous (i.e., concatenated trials). Moreover, data selection may be restricted to one or more specific sleep stage(s), and returned trials may be requested to have a minimum duration. Hence, the user may select e.g. all > 1 min bouts of clean N2 for further analysis. This is further illustrated in Figure 5, where the data quality plot (Figure 5A) shows a large block between 5.5–6 h of the recording marked for rejection, primarily scored as N2 sleep. Correspondingly, the N2 power spectrum of the raw data (i.e., including segments marked for rejection and prior to any channel interpolation) does not show expected sigma peaks (Figure 5B). Rejection of bad segments leads to a large reduction in total power while clear sigma peaks become apparent, though some channels remain noisy (Figure 5C). Finally, spectral appearance further improves following segment-wise data repair via channel interpolation (Figure 5D).

### Application to data

**FIGURE 6:**
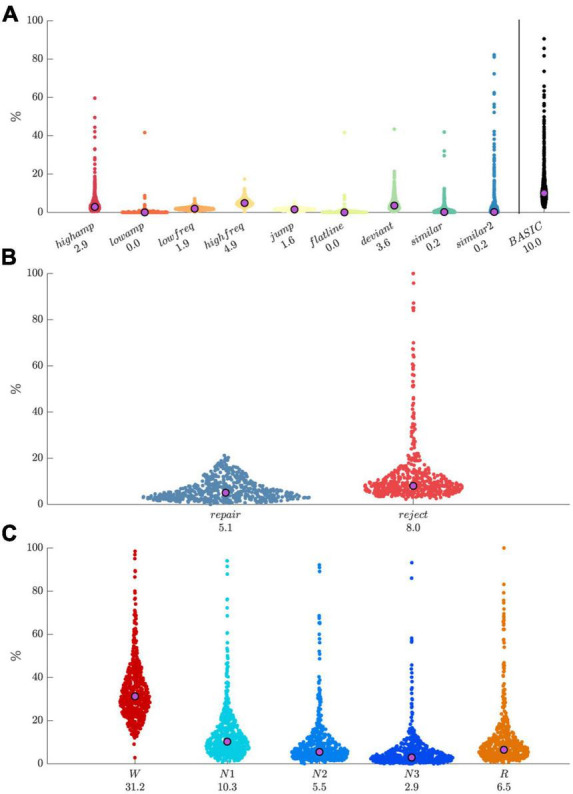
Distributions of artifact percentages and medians across 560 recordings. **(A)** Percentage by artifact type, and their combination (“basic”). **(B)** Repair and reject percentages. **(C)** Percentage of pooled artifacts by sleep stage.

We applied the default detector set to our dataset of 251-channel lab-based recordings (N = 560). For grid-based artifact processing, we set the spatial expansion threshold to 0.75, the segment rejection threshold to 0.25, and the temporal expansion threshold to 0.25. Resulting distributions of artifact percentages by detector type (Figure 6A) had relatively low medians with values < 5%, but were skewed with most detector types flagging some highly anomalous recordings, which was later confirmed by visual review of several recordings. The overlap of all artifact types (basic) yielded a median percentage of 10%. Median percentages of data marked for repair and rejection were around 5% and 8%, respectively, with the rejection distribution being particularly skewed (Figure 6B). Pooling across all detectors, artifact percentages were strongly modulated by sleep stage (Figure 6C), with largest values for W, followed by N1, R, N2, and N3, as might be expected. Note that all preceding values depend importantly on included detectors and their thresholds, as well as parameters for grid-based artifact processing.

### Comparison to manual artifact annotation

For comparison purposes, artifacts were manually annotated by an experienced sleep EEG researcher (RC) for three 18-channel recordings. Annotation was done channel- and segment-wise (i.e., similar to the automated approach) and blindly (i.e., without reference to automatically detected events). Criteria were subjective: a channel-segment element was labeled artifactual whenever the rater deemed the signal too noisy or non-physiological for standard sleep EEG analyses. Exceptions were made for eye blinks during wakefulness and eye movements during REM, since the default automated approach was not set up for these particular events either. Annotating a single 18-channel recording in this manner took about 8 h. Considering manual annotations as the reference, Table 3 details performance of the automated approach using several metrics. Score ranges were high for accuracy (0.92–0.95), sensitivity (0.79–0.90), and specificity (0.93–0.95). However, as the automated approach labeled approximately twice as many channel-segment pairs as artifactual relative to manual annotation, scores were lower for Cohen’s kappa (0.51–0.60), precision (0.39–0.48), and F1 score (0.53–0.62).

**TABLE 3:** Comparison of manual and automatic artifact detection.

Recording	N	Prevalence manual	Prevalence automatic	Accuracy/Agreement	Cohen’s kappa	Sensitivity (tpr)	Specificity (tnr)	Precision (ppv)	F1 score
1	111456	0.046	0.088	0.950	0.600	0.904	0.952	0.476	0.624
2	122904	0.036	0.076	0.947	0.505	0.816	0.952	0.392	0.529
3	102060	0.077	0.129	0.916	0.551	0.791	0.927	0.476	0.594

N, number of channel-segment elements; TPR, true positive rate; TNR, true negative rate; PPV, positive predictive value.

### Pre-cleaning ICA

The default detector set detects many visually apparent artifacts, but other events, such as certain eye- and heart-related waveforms, are not routinely recognized. While this could potentially be addressed by optimizing or adding detectors, the ICA technique is another useful approach for reducing such artifacts, which should typically be done prior to regular artifact detection and data repair. SleepTrip’s ICA approach relies on the same Infomax module used by EEGLAB and FieldTrip, but offers various additional options to make it more suitable for sleep EEG.

First, the user designates the sleep stages that should be included for ICA training. Although ICA can be run on an entire recording in principle, this can be unnecessarily time-consuming. An alternative approach is to run ICA exclusively on the sleep stages most likely to contain artifact (recommended: W, N1, R). Later, selected artifactual components may be subtracted from the entire recording, often with qualitative improvements even for stages not part of the training data (i.e., N2, N3). Second, to prevent ICA from allocating valuable components to extremely noisy data segments or channels, the data supplied to the ICA algorithm may itself undergo some pre-cleaning. Specifically, the default *highamp* and *deviant* detectors are used to a) identify and interpolate anomalous channels and b) identify and remove noisy segments from the data. Third, Infomax *runica* is run, including optional initial PCA (principal component analysis) to reduce the number of returned components. The PCA option is also automatically invoked when the data are not full rank (i.e., incomplete linear independence between channels), which is always the case when channels have been interpolated. Fourth, returned components are automatically classified using the IClabel functionality (Pion-Tonachini et al., 2019) along with classification probabilities. Fifth, the user manually specifies components to remove from the data. Runtimes for ICA decomposition of 251-channel data are approximately 4 h when using the full recording and ∼45 min when limited to stages W, N1 and R.

**FIGURE 7:**
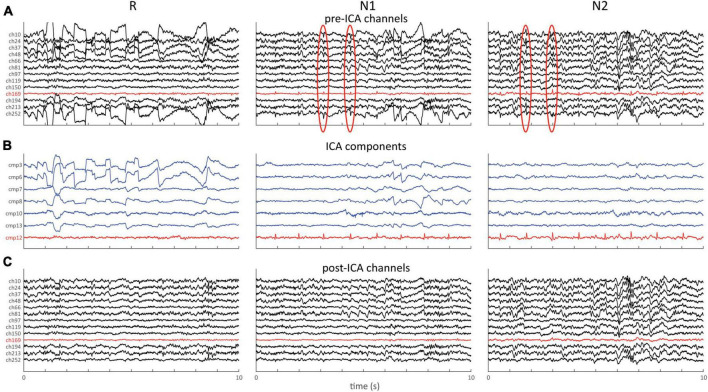
ICA example showing 10 s of data for stages R, N1, and N2. **(A)** Pre-ICA channel data for selection of channels. Note clear ocular activity on several channels during R and N1, and cardiac activity during N1 and N2 (red channel and red ovals). **(B)** Selection of components capturing ocular (blue) and cardiac (red) activity. **(C)** Same channels as in **(A)** following removal of the 7 components shown in **(B)**.

Figure 7 shows an example of the aforementioned approach applied to 64-channel data, with the ICA algorithm being trained exclusively on stages W, N1 and R. Raw channel data (Figure 7A) shows clear ocular activity during R and N1, as well as subtle repetitive cardiac-related activity during N1 and N2 (red channel and ovals). The latter may be easily missed as it is obscured on most channels by higher amplitude brain activity. ICA decomposition (Figure 7B) returns several components capturing these eye- (blue) and heart-related (red) activities, which may be subtracted from the original data (Figure 7C). Clear improvements in signal quality are apparent, including the removal of cardiac activity from N2, even though the ICA algorithm was never exposed to data from this stage. We note that numerous artifacts typically remain after this process, which could then be handled using the previously described cleaning process.

## Discussion

This paper introduces an efficient and flexible approach to automated cleaning of multichannel sleep EEG. Core features include the ability to detect and repair artifacts channel-wise, accommodate artifacts of various types, customize and extend detection options, and visualize detected events and overall data quality. In addition, pre-cleaning ICA may further enhance data quality for high-density recordings. Importantly, once final parameter settings have been selected, cleaning may proceed without user intervention.

### Artifact detection and repair

While many specialized algorithms exist for wake EEG artifact detection and/or repair (Jiang et al., 2019), approaches geared specifically towards sleep EEG with its unique physiology are much less prevalent (’t Wallant et al., 2016; Saifutdinova et al., 2019; Leach et al., 2023). Regardless of wake/sleep state, many proposed signal-processing algorithms make unreasonable computing demands when faced with long-duration and/or high-density EEG recordings, both in terms of memory usage and runtime. In addition, most available sleep cleaning tools have a restricted focus, such as handling only a limited number of artifact types, detection without visualization, detection without repair functionality, minimum/maximum channel counts, and so on. Consequently, it is often left to the user to select, reimplement, and integrate different tools into a coherent cleaning approach. These limitations are shared by various high-level toolboxes (e.g., FieldTrip, EEGLAB, MNE Python), which include powerful artifact-related functionality in the context of wake EEG, but require considerable and often advanced user effort to make them minimally suitable for sleep EEG. The current approach addresses these issues by integrating several convenient data cleaning, repair, and visualization functions into the SleepTrip platform, thereby offering the tools for setting up a complete and customizable pipeline for data handling, preprocessing, cleaning, and analysis.

Default artifact detectors and their settings were chosen following extensive visual examination of individual events, data quality plots, and spectral plots across several dozen lab-based 251-channel EEG recordings (and lower-density variants). Here, particular attention was paid to both the detection of visually apparent artifacts, and the non-detection of neural events at risk of being flagged (e.g., large slow waves/K-complexes). Although provided defaults are deemed reasonable for our data, qualitative detection performance still varied across individual recordings. Consequently, it is likely that the default detector set continues to evolve. For example, each default detector is currently set up to be applied to the entire recording, whereas stage-specific parameter settings might lead to improved specificity and sensitivity. While our focus during software development and in this paper has been on high-density EEG, the approach also appears to work well on low-density or even single-channel EEG (though this was not systematically evaluated).

More broadly, we emphasize that default cleaning settings are intended as a useful starting point rather than an out-of-the-box solution to blindly apply to new data. Different study populations (e.g., clinical, pediatric), recording setups (e.g., cap, amplifier), and data properties (e.g., channel number and location, reference location, sample rate, preprocessing steps) likely necessitate different detectors and parameter settings. For example, amplitude thresholds set in μV may need to be altered depending on reference location. Similarly, thresholds based on z-scores require consideration of the underlying data: for example, the minimum z-score for low-frequency artifacts needs to be comparatively high for an overnight recording rich in slow waves in order to avoid false positive detection of slow waves, but could be lowered for an afternoon nap without slow waves. Moreover, detection parameters should consider analysis goals. For instance, topographical analyses may require relatively strict segment rejection thresholds to allow meaningful interpolation for the remaining non-rejected segments, whereas other analyses might proceed as long as a minimal number of useable channels are available. Finally, it deserves mention that certain types of events, such as rapid eye movements and arousals, may be considered artifacts for some purposes (e.g., spectral analysis), but in other cases could be targets of investigation that should not be removed. While not formally analyzed in the current work, some of the default detectors return events largely overlapping with eye movements or arousals, suggesting that detectors (or entire detector sets) may be optimized to flag various event types of interest. Hence, beyond custom artifact processing optimized to specific datasets and analysis goals, the current approach may also enable the detection and handling of other (non-artifactual) event types.

The ability to set up data cleaning without user intervention is an important feature of the current approach, particularly as large-volume data analyses become more commonplace (Purcell et al., 2017; Kozhemiako et al., 2022). However, it is up to the user to decide whether and when fully automated cleaning is desirable. In particular, for limited datasets it may be feasible to visually review and adjust detection settings on a per-recording basis. In contrast, larger datasets may be more conveniently handled by automated cleaning supported by strategically placed visual evaluation. A potential workflow for cleaning a new dataset might be: i) apply default settings to several example recordings and perform detailed inspection of detected events, ii) adjust settings until detection performance for example recordings is deemed adequate, iii) review data quality plots and summary tables across full sample to identify outliers for additional detailed inspection, iv) repeat preceding steps until satisfied. While the process of detector building, parameter tweaking, and visual exploration still requires a substantial time investment, in most cases with larger datasets this will still be more time-efficient than manual cleaning. Moreover, performing visual examinations instills confidence that subsequent automated cleaning results will be acceptable. Finally, automated cleaning offers the benefits of reproducibility and makes it possible to examine the impact of different cleaning strategies on final results (e.g., do results using stricter versus more lenient parameter settings converge?).

We elected not to formally validate the current approach against human detection, as this would require extensive manual channel- and segment-wise artifact annotation, ideally across many high-density recordings. Moreover, given that such detailed annotations are rarely, if ever, performed in the course of typical manual cleaning, it is unclear whether they would serve as a meaningful ground truth. Nevertheless, in order to provide a rough indication of performance, we manually annotated three recordings for comparison with the default automated approach. Relative to manual annotation, the automated approach detected about twice as many artifacts. However, upon visual review this higher artifact prevalence did not seem unreasonable. Additionally, alignment between automatic and manual artifact detection can be improved rather trivially by relaxing automatic detection thresholds. Given the ultimately inherent ambiguity regarding what constitutes a true artifact, we consider the performance values of Table 3 adequate. We did not compare the present approach to other (semi-)automatic sleep EEG cleaning tools (’t Wallant et al., 2016; Saifutdinova et al., 2019; Leach et al., 2023), mainly because these tools do not generate the channel-resolved artifact information required for direct comparison (but see the approach by Leach and colleagues for epoch-wise marking of bad channels). Consequently, it is not claimed that the current approach performs better than manual or other automated approaches. However, we emphasize that the current tool’s primary contribution lies in providing the means to efficiently and flexibly prepare large datasets for analyses, rather than establishing performance metrics for a specific combination of detectors, parameter settings, and data characteristics.

Data repair presently occurs through weighted spherical spline-based interpolation (Freeden, 1984; Oostenveld et al., 2011) or replacement with a fixed value (not-a-number or zero), though other interpolation or repair approaches may be added in the future. While having different channels interpolated at each segment has drawbacks (e.g., different data rank at each segment, small residual discontinuities at segment edges), these concerns are preferred over the data loss that occurs with conventional interpolation across the entire recording. Currently implemented detection options are limited to relatively straightforward low-level signal processing steps (e.g., filter, Hilbert transform, rectify, z-score, signal gradient, correlation) in conjunction with simple detection criteria (e.g., minimum/maximum amplitude and duration thresholds). Indeed, we have deliberately avoided algorithmic steps that make unrealistic computational demands in the context of multichannel sleep EEG, even if they could assist artifact detection in principle. It is currently not possible to add or remove individual artifact events based on visual exploration, as this would require substantial new interactive functionality. While this and other functionality may be added in the future, currently available signal processing options, combined with the various grid-based processing options and repair/selection routines, will likely accommodate a large part of custom cleaning wishes.

### ICA

Given ICA’s proven utility in reducing various artifact types that are otherwise difficult to repair, SleepTrip also includes ICA functionality, which should typically be applied prior to standard artifact detection. While the low-level algorithm is identical to that offered by e.g. FieldTrip and EEGLAB, several high-level options particularly useful to sleep EEG are available. First, a simplified version of the artifact detection and repair functionality is run prior to ICA to avoid supplying overly noisy data to the ICA algorithm. Second, ICA training may be restricted to specific sleep stages, reducing computational burden while still allowing artifact removal across the entire recording.

Several caveats regarding pre-cleaning ICA should be mentioned. First, the assumption of data stationarity does not hold for sleep EEG with its distinct substates, even when excluding particular sleep stages from the ICA algorithm. Second, it is often recommended for wake EEG to high-pass filter data at 1 Hz to prevent ICA’s attention from being drawn to slow dynamics and wasting valuable components on them. However, this suggestion is difficult to follow in the context of sleep EEG where it would remove substantial parts of physiological slow wave and delta activity, complicating visual assessment of returned components. Third, component removal reduces data rank, meaning that any downstream channel interpolation - as part of standard data repair – represents a second round of rank reduction. Despite these considerations, the implemented ICA approach often returns clearly artifactual components that, when subtracted from the original data, yield substantial improvements in signal appearance (Figure 7). In light of our overall objective of improving data quality for subsequent analyses, we deem these deviations from recommended practices acceptable and in line with previous sleep ICA approaches (Romero et al., 2003; Crespo-Garcia et al., 2008; Demanuele et al., 2017). We emphasize that it is up to the user whether specific components should be considered artifactual and/or removed from the data, as components could also be used to directly quantify features of interest (e.g., rapid eye movements, heart rate or its variability).

For convenience, ICA components are returned with probabilities of belonging to each of seven classes (brain, muscle, eye, heart, line noise, channel noise, other), as provided by the IClabel functionality (Pion-Tonachini et al., 2019). IClabel provides classification information by comparing components to templates, which in turn are based on human annotation of many thousands of components. Importantly, however, IClabel’s templates are trained exclusively on wake data, and classification of sleep EEG components is therefore far from perfect. Nonetheless, labeling of ocular and cardiac components often appears quite reasonable and may assist manual selection of components to remove. While class probabilities could be used to automate component removal in principle, preliminary efforts suggest that manual component selection leads to better results and is the recommended approach for the time being.

## Conclusion

This paper introduces an approach for customizable automated detection and repair of multichannel sleep EEG artifacts, integrated in a widely used framework for EEG analysis. This tool may help improve sleep EEG cleaning practices and could serve as a basis for creating standardized cleaning pipelines, ultimately accelerating fundamental and clinical sleep EEG research.

## Data availability statement

SleepTrip data is available at GitHub: https://github.com/coxroy/sleeptrip. A Wiki is available at https://github.com/coxroy/sleeptrip/wiki. Tutorial cleaning scripts are included in SleepTrip; accompanying tutorial data are available from Zenodo: https://zenodo.org/doi/10.5281/zenodo.10256036. The full dataset used for analysis in this study is not publicly available due to its large size, further enquiries should be directed to RC, roycox.roycox@gmail.com.

## Ethics statement

The studies involving humans were approved by the VU Medical Center or University of Amsterdam. The studies were conducted in accordance with the local legislation and institutional requirements. The participants provided their written informed consent to participate in this study.

## Author contributions

RC: Conceptualization, Formal analysis, Methodology, Software, Visualization, Writing–original draft, Writing–review and editing. FW: Conceptualization, Methodology, Writing–review and editing, Software. EV: Conceptualization, Funding acquisition, Methodology, Writing–review and editing, Resources, Supervision.

## Footnotes

1
https://github.com/coxroy/sleeptrip

2
https://zenodo.org/doi/10.5281/zenodo.10256036

## References

[B1] Anderer P. Roberts S. Schlögl A. Gruber G. Klösch G. Herrmann W. (1999). Artifact processing in computerized analysis of sleep EEG – a review. *Neuropsychobiology* 40 150–157. 10.1159/000026613 10494051

[B2] Attarian H. P. Undevia N. S. (2012). *“Artifacts”: Atlas of electroencephalography in sleep medicine.* Boston, MA: Springer US, 69–92. 10.1007/978-1-4614-2293-8_5

[B3] Belonosov G. Falach R. Schmidig J. F. Aderka M. Zhelezniakov V. Shani-Hershkovich R. (2023). SleepEEGpy: A python-based package for the preprocessing, analysis, and visualization of sleep EEG data. *bioRxiv* [Preprint]. 10.1101/2023.12.17.572046 40288293

[B4] Berry R. B. Albertario C. L. Harding S. M. Lloyd R. M. Plante D. T. Quan S. F. (2018). *AASM scoring manual version 2.5. 90.* American Academy of Sleep Medicine.

[B5] Cox R. Fell J. (2020). Analyzing human sleep EEG: A methodological primer with code implementation. *Sleep Med. Rev.* 54:101353. 10.1016/j.smrv.2020.101353 32736239

[B6] Crespo-Garcia M. Atienza M. Cantero J. L. (2008). Muscle artifact removal from human sleep EEG by using independent component analysis. *Ann. Biomed. Eng.* 36 467–475. 10.1007/s10439-008-9442-y 18228142

[B7] Delorme A. Makeig S. (2004). EEGLAB: An open source toolbox for analysis of single-trial EEG dynamics including independent component analysis. *J. Neurosci. Methods* 134 9–21. 10.1016/j.jneumeth.2003.10.009 15102499

[B8] Demanuele C. Bartsch U. Baran B. Khan S. Vangel M. G. Cox R. (2017). Coordination of slow waves with sleep spindles predicts sleep-dependent memory consolidation in schizophrenia. *Sleep* 40:zsw013. 10.1093/sleep/zsw013 28364465 PMC6084745

[B9] Freeden W. (1984). Spherical spline interpolation—basic theory and computational aspects. *J. Comput. Appl. Maths.* 11 367–375. 10.1016/0377-0427(84)90011-6

[B10] Gramfort A. (2013). MEG and EEG data analysis with MNE-python. *Front. Neurosci.* 7:267. 10.3389/fnins.2013.00267 24431986 PMC3872725

[B11] Hermans L. W. A. Huijben I. A. M. van Gorp H. Leufkens T. R. M. Fonseca P. Overeem S. (2022). Representations of temporal sleep dynamics: Review and synthesis of the literature. *Sleep Med. Rev.* 63:101611. 10.1016/j.smrv.2022.101611 35278893

[B12] Jiang X. Bian G.-B. Tian Z. (2019). Removal of artifacts from EEG signals: A review. *Sensors* 19:987. 10.3390/s19050987 30813520 PMC6427454

[B13] Kozhemiako N. Mylonas D. Pan J. Q. Prerau M. J. Redline S. Purcell S. M. (2022). Sources of variation in the spectral slope of the sleep EEG. *Eneuro* 9 1–20. 10.1523/ENEURO.0094-22.2022 36123117 PMC9512622

[B14] Leach S. Sousouri G. Huber R. (2023). ‘High-density-SleepCleaner’: An open-source, semi-automatic artifact removal routine tailored to high-density sleep EEG. *J. Neurosci. Methods* 391:109849. 10.1016/j.jneumeth.2023.109849 37075912

[B15] Oostenveld R. Fries P. Maris E. Schoffelen J.-M. (2011). FieldTrip: Open source software for advanced analysis of MEG, EEG, and invasive electrophysiological data. *Comput. Intell. Neurosci.* 2011:156869. 10.1155/2011/156869 21253357 PMC3021840

[B16] Pion-Tonachini L. Kreutz-Delgado K. Makeig S. (2019). ICLabel: An automated electroencephalographic independent component classifier, dataset, and website. *Neuroimage* 198 181–197. 10.1016/j.neuroimage.2019.05.026 31103785 PMC6592775

[B17] Pisarenco I. Caporro M. Prosperetti C. Manconi M. (2014). High-density electroencephalography as an innovative tool to explore sleep physiology and sleep related disorders. *Int. J. Psychophysiol.* 92 8–15. 10.1016/j.ijpsycho.2014.01.002 24412343

[B18] Purcell S. M. Manoach D. S. Demanuele C. Cade B. E. Mariani S. Cox R. (2017). Characterizing sleep spindles in 11,630 individuals from the national sleep research resource. *Nat. Commun.* 8:15930. 10.1038/ncomms15930 28649997 PMC5490197

[B19] Rechtschaffen A. Kales A. (1968). *A manual of standardized terminology, techniques and scoring system for sleep stages of human subjects.* Washington, DC: Public Health Service, US Government Printing Office.

[B20] Romero S. Mananas M. A. Clos S. Gimenez S. Barbanoj M. J. (2003). “Reduction of EEG artifacts by ICA in different sleep stages,” in *Proceedings of the 25th annual international conference of the IEEE engineering in medicine and biology society (IEEE Cat. No.03CH37439)*, (Cancun: IEEE), 2675–2678. 10.1109/IEMBS.2003.1280467

[B21] Saifutdinova E. Congedo M. Dudysova D. Lhotska L. Koprivova J. Gerla V. (2019). An unsupervised multichannel artifact detection method for sleep EEG based on riemannian geometry. *Sensors* 19:602. 10.3390/s19030602 30709001 PMC6387048

[B22] ’t Wallant D. C. Muto V. Gaggioni G. Jaspar M. Chellappa S. L. Meyer C. (2016). Automatic artifacts and arousals detection in whole-night sleep EEG recordings. *J. Neurosci. Methods* 258 124–133. 10.1016/j.jneumeth.2015.11.005 26589687

[B23] Tadel F. Baillet S. Mosher J. C. Pantazis D. Leahy R. M. (2011). Brainstorm: A user-friendly application for MEG/EEG analysis. *Comput. Intell. Neurosci.* 2011 1–13. 10.1155/2011/879716 21584256 PMC3090754

[B24] Weber F. D. Supp G. G. Klinzing J. G. Mölle M. Engel A. K. Born J. (2021). Coupling of gamma band activity to sleep spindle oscillations – a combined EEG/MEG study. *Neuroimage* 224:117452. 10.1016/j.neuroimage.2020.117452 33059050

