# Supplementary material for: Customizable automated cleaning of multichannel sleep EEG in SleepTrip
Source: Front Neuroinform. 2024 Aug 9;18:1415512. doi: 10.3389/fninf.2024.1415512 (PMC11341374; doi:10.3389/fninf.2024.1415512)
Supplement: Supplementary file 1 [file Data_Sheet_1.PDF]

Customizable automated cleaning of multichannel sleep EEG in SleepTrip

Supplementary Materials

Roy Cox, Frederik D Weber, Eus JW Van Someren

Supplementary figures 1-6 show various data quality plots from very clean to very noisy.

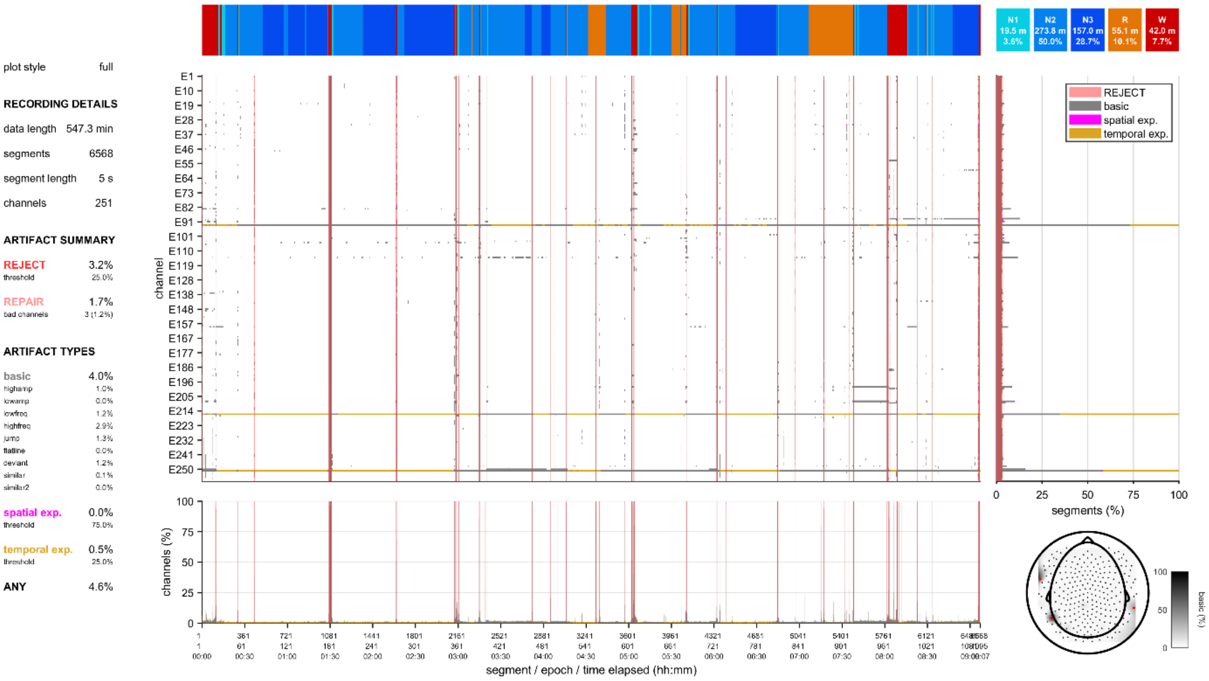

Supplementary Figure 1

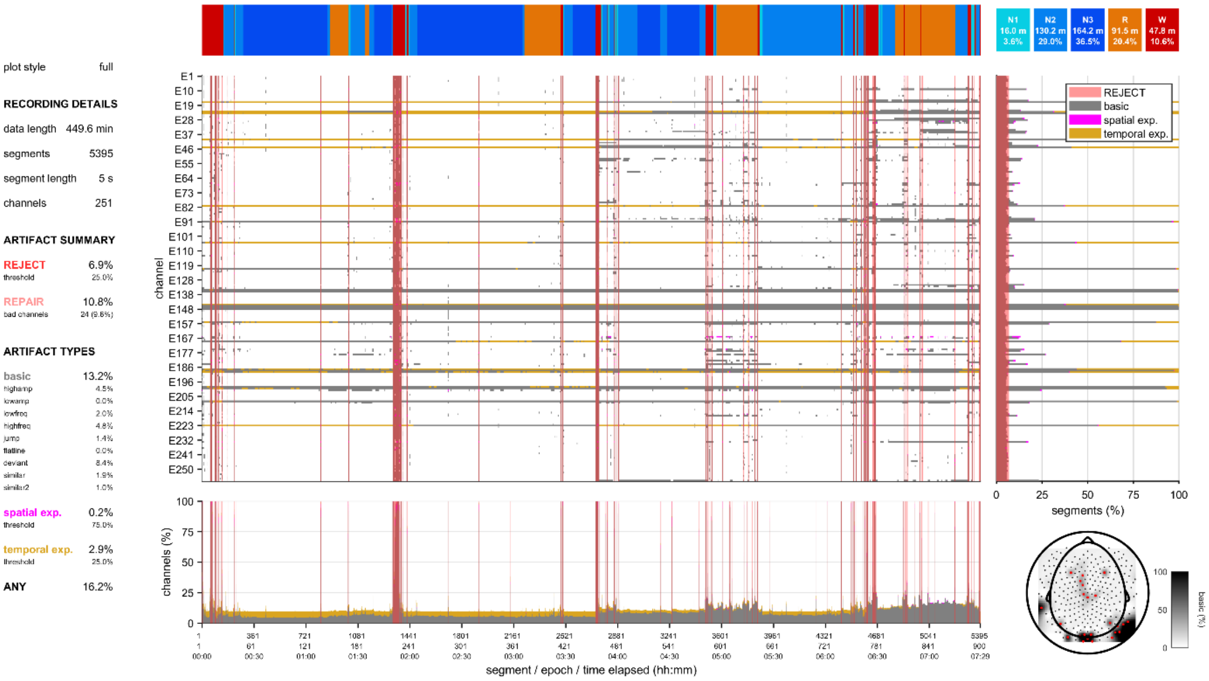

Supplementary Figure 2

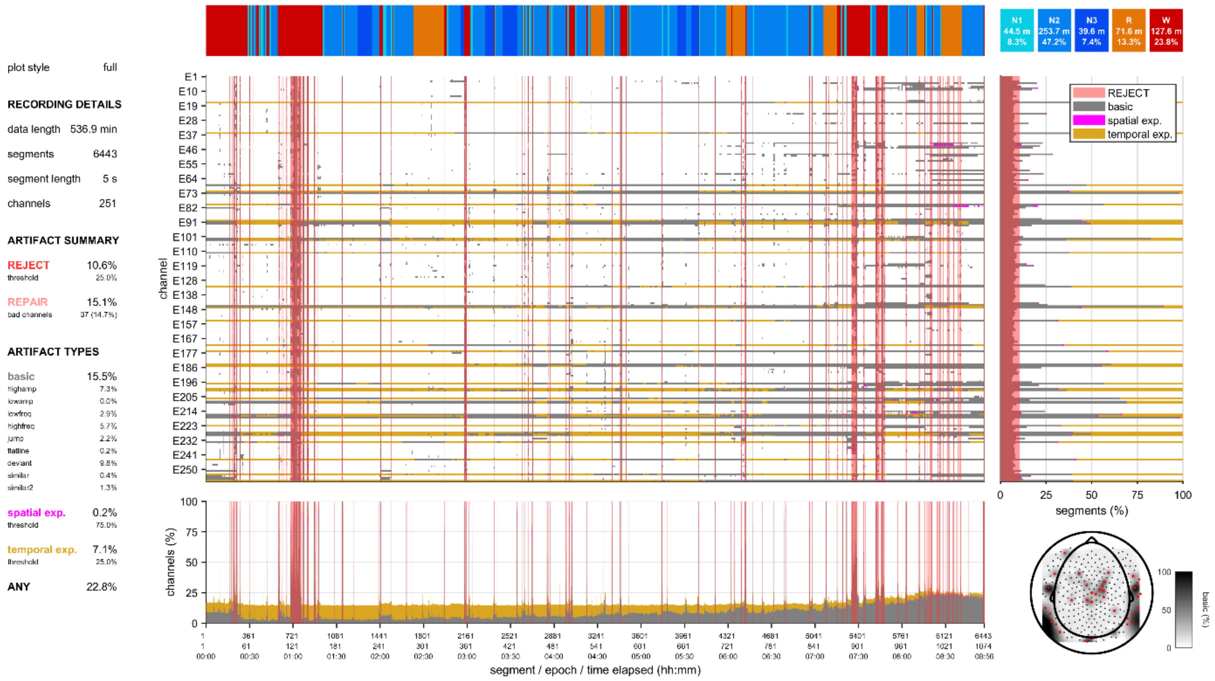

Supplementary Figure 3

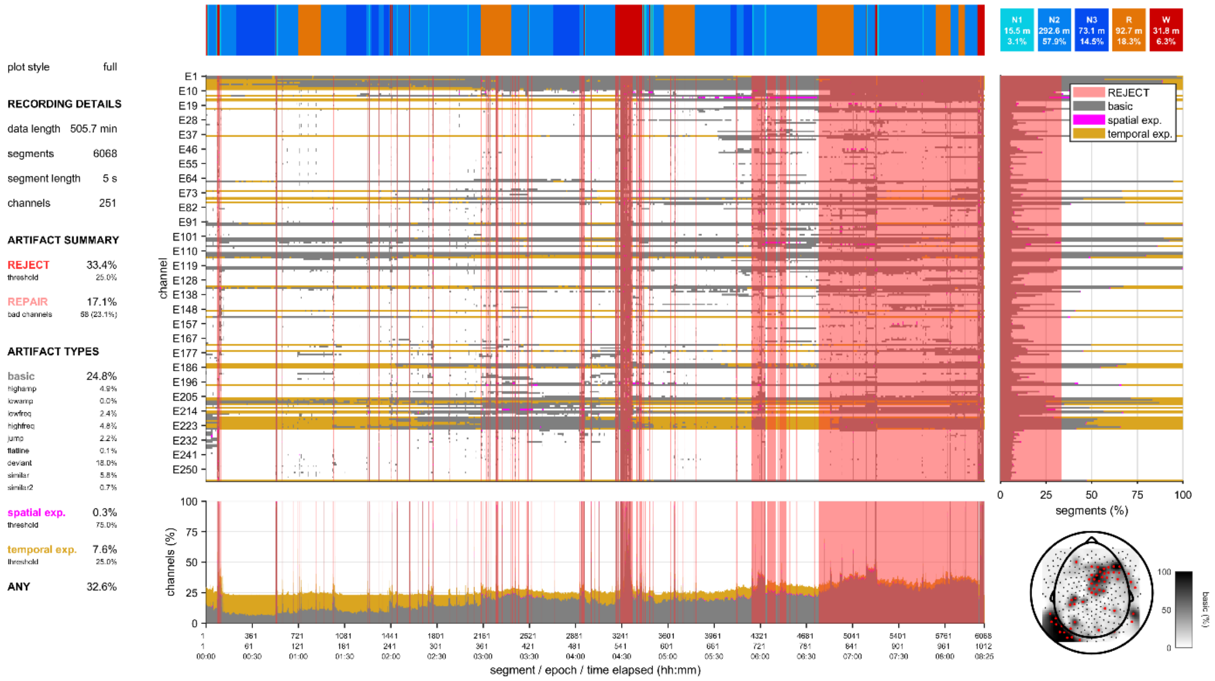

Supplementary Figure 4

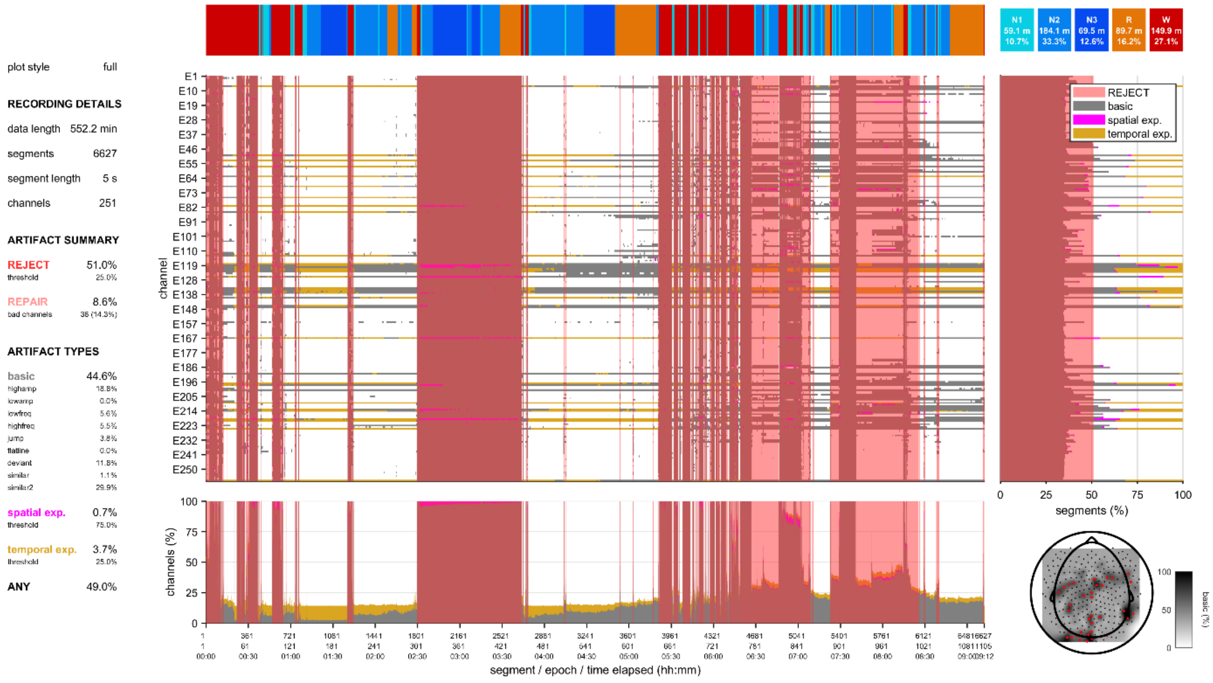

Supplementary Figure 5

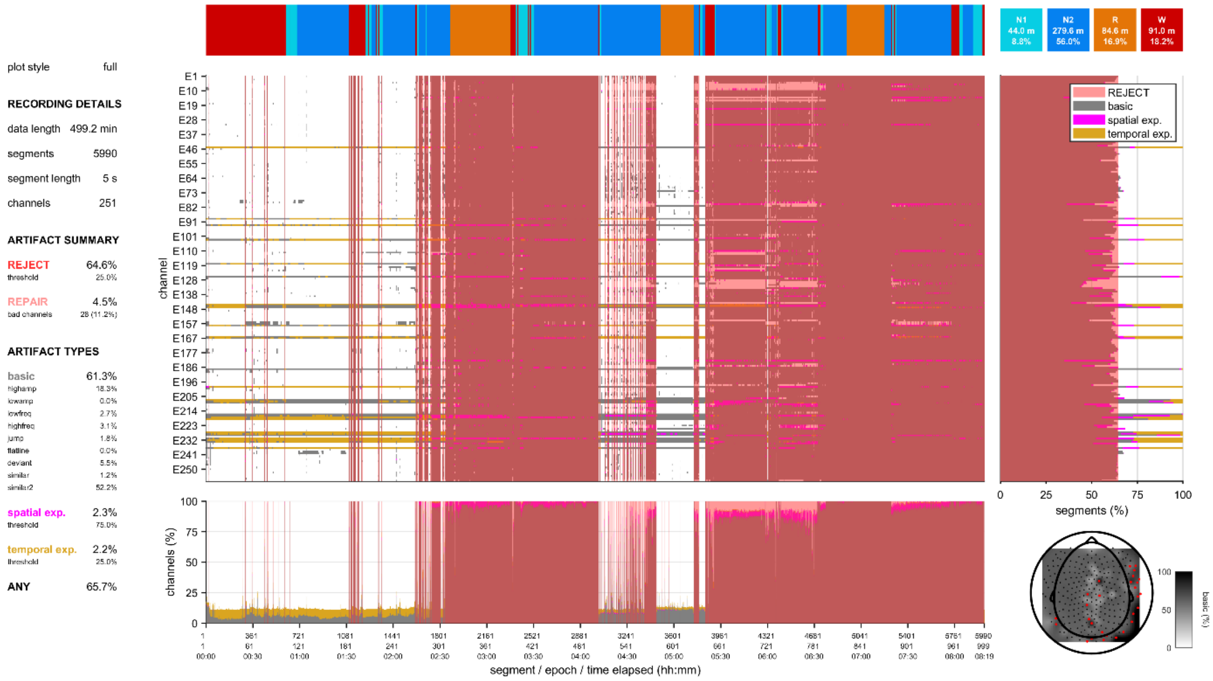

Supplementary Figure 6
